# Editorial: Smart cities challenges, technologies and trends

**DOI:** 10.3389/fdata.2023.1258051

**Published:** 2023-10-17

**Authors:** Namita Gupta

**Affiliations:** Maharaja Agrasen Institute of Technology, New Delhi, India

**Keywords:** smart cities trends, smart cities challenges, smart cities technologies, societal challenges, smart city developments

We are delighted to announce the publication of the Frontiers' Research Topic titled “Smart cities challenges, technologies and trends” organized in collaboration with Department of Computer Science and Engineering, Maharaja Agrasen Institute of Technology and National Institute of Technology Delhi, the organizers of the 2022 International Symposium on Smart Cities Challenges, Technologies, and Trends (SCCTT-2022). This peer-reviewed article Research Topic aims to raise awareness about the role of smart cities in the future, emphasizing their intelligent infrastructure, modern architecture, efficient communication, and citizen-friendly services. The Research Topic, accessible through Frontiers' open science platform and searchable across multiple journals, offers insights into the latest trends and challenges in Smart Cities technology, fostering collaboration to develop cost-effective solutions for urban issues such as traffic congestion, energy consumption, and inadequate infrastructure. However, the journey toward building smart cities is not without obstacles.

This editorial preface provides an overview of the challenges, technologies, and trends that shape the landscape of smart cities.

## Challenges

Implementing smart city initiatives involves overcoming several challenges including the integration of diverse technologies and legacy infrastructure, ensuring data privacy and cybersecurity, fostering citizen engagement and participation in decision-making, addressing the digital divide to provide equal access to services, managing sustainable and energy-efficient practices, navigating complex regulatory frameworks, securing sufficient funding and investments, and developing resilient strategies to handle potential disasters and emergencies. These challenges require collaborative efforts and innovative solutions to create efficient, inclusive, and sustainable urban environments for the future.

## Technologies

Smart cities rely on various cutting-edge technologies to optimize urban operations and enhance quality of life. The Internet of Things forms the foundation, enabling real-time data collection and analysis through connected devices and sensors. Big data analytics extracts valuable insights from the vast amounts of data generated, facilitating informed decision-making, and optimized services. Artificial Intelligence automates processes and optimizes resource allocation, improving efficiency and personalizing citizen services. AI also reduces network costs, enhances performance, and enables a wide range of telecom services and applications in smart city networks. While technology undoubtedly plays a significant role in smart city developments, it is essential for all stakeholders to consider the broader societal impact. Alongside implementing state-of-the-art technology, having a clear mission, and measuring the effects on the environment, society, and governance are crucial for achieving long-term sustainability. A human-centric approach and design thinking are vital to enhance the quality of life for smart city residents.

## Trends

Smart cities are witnessing several emerging trends that shape their future. Sustainable energy management is a key focus, with a shift toward renewable sources, energy-efficient buildings, and smart grids. Among the prevailing trends, smart mobility encompasses intelligent traffic management, eco-friendly transportation, autonomous vehicles, and car-sharing initiatives. Additionally, tech solutions for digital citizens are gaining momentum, enabling remote healthcare, interactive education, and entertainment. Startups are also leveraging blockchain to enhance safety and security measures for residents. Furthermore, progress in robotics, extended reality, and drones is empowering urban planning, agriculture, and waste management practices. These trends contribute to reducing carbon emissions, improving transportation systems, and fostering inclusivity in smart city development.

Total six articles are published on the given Research Topic. Summary of each article is as follows:

Singh and Ujjwal on Topic “Hybridized bio-inspired intrusion detection system for Internet of things”: A hybridized bio-inspired intrusion detection system for Smart Cities utilizes advanced algorithms inspired by biological systems to detect and respond to security threats, enhancing the overall safety and resilience of urban environments. In this article, authors propose a hybrid bio-inspired-based intrusion detection system (IDS) for the IoT framework, using the sine-cosine algorithm (SCA) and salp swarm algorithm (SSA) to determine network traffic features. Selected features are passed to a machine learning (ML) classifier for the detection and classification of intrusive traffic.

Nagvanshi et al. on Topic “Nonstationary time series forecasting using optimized-EVDHM-ARIMA for COVID-19”: The optimized-EVDHM-ARIMA approach leverages an evolutionary algorithm and the Ensemble Variational Mode Decomposition for nonstationary time series forecasting in both COVID-19 data and Smart Cities applications, enabling accurate predictions and proactive decision-making for public health and urban management. Here, Authors present a forecasting model for COVID-19 infections which combines multiple components forecasted by ARIMA and utilizes genetic algorithms to optimize parameter selection and decomposition results, providing valuable insights into virus transmission rate and future infection cases for effective decision-making and healthcare preparedness.

Singh et al. on Topic “Blockchain-enabled access control to prevent cyber-attacks in IoT”: Blockchain-enabled access control provides a decentralized and tamper-proof mechanism for managing user permissions and data access in Smart Cities, reducing the risk of cyber-attacks by preventing unauthorized entry and ensuring data security and integrity across interconnected systems and devices.

Duggal et al. on Topic “Dependable modulation classifier explainer with measurable explainability”: It employs interpretable machine learning techniques to elucidate the decision-making process behind modulation classification in wireless communication systems. By quantifying feature importance, model confidence, and providing human-understandable explanations, it ensures transparency and accountability, enabling stakeholders to assess and validate the system's performance and dependability in critical Smart City applications. The proposed dependable modulation classifier explainer (DMCE) focuses on the explainability of modulation classification, allowing for visualization and understanding of prediction results. The study presents a comparative analysis with existing methods and demonstrates the potential of deep learning in enhancing IoT network performance.

Sharma et al. on Topic “An extended approach to appraise electricity distribution and carbon footprint of bitcoin in a smart city”: This study explores the energy consumption and carbon emissions associated with Bitcoin mining. It extends the Patch methodology by considering hardware efficiency limits and power generation sources, providing a more refined estimate of electricity consumption and carbon footprint. The study estimates that Bitcoin consumed between 38.495 and 120.72 terawatt hours of electricity in 2021 and released between 2.12 and 45.37 million metric tons of carbon dioxide. Efforts to address energy consumption include the relocation of miners to energy-intensive regions, such as aluminum mining sites powered by hydroelectricity.

Shambharkar and Goel on Topic “From video summarization to real time video summarization in smart cities and beyond: A survey”: This article discusses the importance of video summarization in managing and retrieving large volumes of video data, particularly in the context of smart cities and surveillance systems. It presents an analysis of video summarization approaches, focusing on real-time video summarization techniques. The study aims to provide valuable insights for researchers and practitioners, highlighting the practical applications of video summarization in smart cities, such as aberrant detection in video surveillance systems.

In summary, the construction of smart cities is a complex and ambitious endeavor. Despite the significant challenges associated with infrastructure upgrades, data privacy and security, and interoperability, the potential rewards are substantial. Smart cities have the potential to improve efficiency, sustainability, and the overall quality of life by harnessing technologies like the integration of the Internet of Things, artificial intelligence, cloud computing, and data analytics to support sustainable transformations across various city components. Notably, the prevailing trend in smart cities is smart mobility, covering intelligent traffic management, zero-emission transportation, autonomous vehicles, and car-sharing programs. Tech solutions such as remote healthcare, interactive education, and entertainment, are also gaining momentum. To ensure successful smart city developments, stakeholders must prioritize purposeful missions, measuring environmental and societal impacts, and embrace design thinking anchored in human-centric approaches. Encouraging an abundance mindset and empowering younger generations with creative thinking can lead to transformative advancements for the greater good and address long-standing societal challenges.

## Author contributions

NG: Writing—review and editing.

